# Investigation of the Thermal Comfort Properties of Masks Used during the COVID-19 Pandemic

**DOI:** 10.3390/ijerph191811275

**Published:** 2022-09-08

**Authors:** Eren Oner, Ahmet Çağdaş Seçkin, Dilara Egeli, Mine Seçkin

**Affiliations:** 1 Textile Engineering Department, Usak University, Usak 64200, Turkey; 2 Computer Engineering Department, Adnan Menderes University, Aydin 09010, Turkey

**Keywords:** COVID-19 pandemic, mask, thermal comfort, thermal head measurement system

## Abstract

SARS-CoV-2, the causative agent of COVID-19, which was officially declared a pandemic by the World Health Organization (WHO) on 11 March 2020, is transmitted from person to person through respiratory droplets and close contact and can cause severe respiratory failure and pneumonia. Currently, while the worldwide COVID-19 pandemic is still ongoing and countries are taking strict precautions to protect populations against infection, the most effective precautions still seem to be social distancing and wearing a mask. The question of how effective masks were in the early stages of the COVID-19 pandemic has been widely discussed, both in public and scientific circles, and the protection of different mask types has been examined. This study aimed to examine the comfort conditions provided by the different mask types to the user during use. For this purpose, single-ply, double-ply, three-ply, cloth, FFP1, FFP2, and FFP3 masks with different standards were examined, with and without a valve. To conduct the experiments, the novel thermal head measurement system, developed within the scope of this study, was used specifically for mask comfort studies. Thanks to the developed measurement system, the thermal resistance and water vapor resistance values of different masks were measured, and their comfort conditions were evaluated. According to the findings, cloth masks provide a comfortable condition, with lower thermal resistance and water vapor resistance values than other masks. In addition, it was observed that surgical masks offer better thermal comfort conditions, although they have lower protection than FFP masks.

## 1. Introduction

The disease was first identified on 31 December 2019, when it became known that acute respiratory failure syndrome was detected in four individuals working at a seafood and live animal market in Wuhan, Hubei province, China, as well as in numerous individuals visiting that market on the same days. On 7 January, an examination of samples collected from the patients revealed that the virus that caused the illness belonged to the coronavirus family, and the virus was named new coronavirus 2019 (2019-nCoV). The new coronavirus was officially named SARS-CoV-2, and the disease it caused was named COVID-19. SARS-CoV-2, the causative agent of COVID-19, which was officially declared a pandemic by the World Health Organization (WHO) on 11 March 2020, is a zoonotic disease in the coronavirus family. It is transmitted from person to person by droplet infection and close contact and can cause severe respiratory failure and pneumonia [1,2,3]. During this pandemic, approximately 553 million people had been infected and 6 million 300 thousand people had died by the time this article was being written [4]. Unfortunately, the number of infected and dying people continues to increase.

During the COVID-19 pandemic, tools and technologies for the rapid control of highly contagious viruses have been researched and studies have been presented [5,6,7]. Despite all these important studies, at the beginning of the pandemic the use of masks proved to be the simplest and fastest solution. The use of masks is still one of the most important precautions at this time, when the global COVID-19 pandemic is still ongoing, and countries are taking strict precautions to protect themselves from infection. A mask is defined as medical equipment that covers the mouth and nose to create a barrier that minimizes the direct transmission of infectious agents between sick and non-sick individuals [8]. In many studies, the collective wearing of face masks has been found to help control COVID-19 by reducing the emission of infected saliva and respiratory droplets from individuals with subclinical or mild COVID-19 [9,10,11,12,13,14,15]. During the ongoing COVID-19 pandemic, the wearing of face masks has become mandatory in many countries, especially in closed and crowded spaces, public transportation, and workplaces. As Matuschek et al. stated, “the surgical face mask has become a symbol of our times” [16].

The use of face masks has become commonplace and ubiquitous, not only in hospitals but also in society. There are various masks with different protective functions, different designs and different standards. Single-ply, double-ply and three-ply surgical masks, masks made of woven or knitted fabric, masks with particle filter FFPs (filtering face piece) and many different derivatives are widely used in society. These masks, called respiratory masks (RM), are protective equipment that cover part of the face. They are designed to protect both the wearer and the immediate environment from respirable contaminants and pollutants (respiratory toxins or bacterial/viral pathogens) [16]. During the current COVID-19 pandemic, people must live with masks on for hours. In addition, masks must fit tightly to the face to provide better protection by preventing leaks and inhalation of small airborne aerosols. These conditions cause people to feel uncomfortable and to sweat uncomfortably while wearing them. Although many recent studies have focused on aerosol penetration through masks, limited research has been conducted on the thermal comfort of the different mask types that are used for such long periods of time and cause discomfort in humans. Thermal comfort can be considered as the physiology of a clothed person in a climatic environment, or it can include how it alters the physical properties of clothing and the interaction between the human body and its environment [17]. Important parameters in thermal comfort evaluation are thermal resistance and water vapor resistance. These two parameters are used to determine the heat and mass transfer of the textile and to evaluate the thermal comfort condition [18]. Tang et al. investigated the effects of face masks on thermal comfort in a survey study. It was found that more than 70% of the participants found the masks they used uncomfortable [19]. Maniaci et al. evaluated the comfort conditions of masks used by medical personnel using a questionnaire and found that the masks caused discomfort because they interfered with the humidification process of the inhaled air [20]. The pediatric study by Smart et al. evaluated the comfort status of three different N95 masks in children, aged between 8 and 11 years. The children were asked to walk for three minutes and run for three minutes. Their main complaint was that their faces were hot. In addition, subjective discomfort was reported for one of the three masks [21,22]. Gericke et al. investigated the effect of mask design and the fabric’s structural properties on temperature and humidity in the microclimate between the skin and the fabric mask by measuring with a data logger [23]. Zender-Świercz et al. measured the thermal comfort properties of a range of mask types using a thermal manikin. As a result of the study by Zender-Świercz et al., dry masks were found to increase the comfort levels in temperate climate conditions, while wet masks decreased the comfort status [24]. In this study, some face shields were also investigated as masks. In this study, a full body model was used for measurement, and the model had a computer-aided design and a symmetrical structure.

As can be seen from the studies in the literature, although there have been many studies on the medical protective properties of masks, most of them were surveys, regional measurements with a data logger, or a measurement taken using a thermal manikin, on the thermal comfort properties. It is understood that, in these studies, mostly subjective data were collected, or that thermal and humidity values were measured locally and depending on the person. In the application with the thermal manikin, the thermal properties of a very small area were evaluated in a measurement system, which included the entire body. As it is known from previous studies and from the experiences under pandemic conditions, it is clear that masks alone affect the comfort of the body and that such an important parameter should be measured alone and with a mask-related measurement system. Therefore, the development of a portable and modular measurement system, specifically for mask measurement, is an original and innovative contribution to the studies in this field. In this way, a system will be developed to measure the thermal comfort characteristics of the masks used during the pandemic. For this purpose, a novel thermal head system was introduced, and the thermal comfort properties of the most commonly used mask types were investigated in this study. In contrast to the existing empirical studies, this study aims to make the following contributions to the literature:

1. Conduct thermal comfort tests on single-ply, double-ply, three-ply, cloth, FFP1, FFP2, FFP3, and valve mask groups, which were used during the pandemic and are still in use in sectors such as healthcare.
2. The proposed method should be able to determine the thermal comfort characteristics of the masks studied. The thermal resistance and water vapor resistance values were calculated using the system. A reliability analysis of the thermal head model was performed.
3. A 3D-printed and portable thermal head model has been produced for masks, for the first time.

## 2. Materials and Methods

Within the scope of this study, 10 different mask types that were commonly used during the COVID-19 pandemic period, were used in the experiments. The mask types used in the experiments are given in Table 1.

### Measurement Method

In order to examine the thermal comfort properties of masks, a thermal head model was designed and produced specifically for this study. Thus, in this study, the device for measuring the thermal comfort properties of masks was introduced.

**Figure 1:**
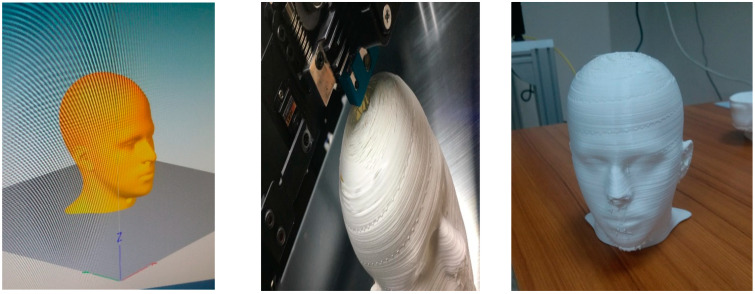
Production steps of the head model.

Firstly, the thermal head system was designed according to a realistic human body size, with SolidWorks software. In order to reflect a real human face, the design was made by scanning real human body sizes. According to the precise design, the parts were processed one-by-one, with the Simplify 3D slicing software. Afterwards, the parts designed and sliced were printed by an industrial-scale 3D printer, Coremax 600 Pro (Figure 1).

**Figure 2:**
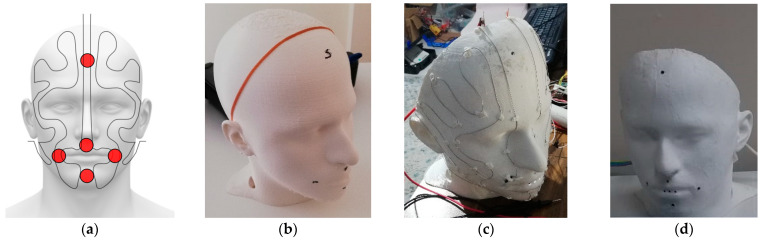
The resistance and sensor application stages on the head model. (**a**) Thermal head resistor and sensor layout. (**b**) The state of the 3D print head model before it is processed. (**c**) Laying of the temporary resistance wire on the head model. (**d**) Resistors embedded in the thermal head model and painted.

The heating system was created by laying resistance wires on the printed thermal head model. Thanks to the high temperature softening of the thermal head model, printed with poly lactic acid (PLA) polymer, resistance wires were applied over the surface, as shown in Figure 2. The placement of the applied resistance wire and head sensors is shown in Figure 2a. In Figure 2b, the placement on the head and the state of the head before cutting are shown. Figure 2c shows the temporary assembled state of the resistance wires. Figure 2d shows the final state of the head model. During this application, first, the wire was mounted temporarily for placement, and then it was heated and embedded in the PLA surface. Heating resistance wire of 0.5 mm thickness was laid at 2 cm intervals. In the head model, the face was considered as two separate halves, vertically, and 1.5 m long and 0.5 mm diameter resistance wire was laid on each half. The resistance value of this resistor wire was approximately 15 ohms. When a 12 V, a 40 A direct current power supply was applied to the resistor wire. It was observed that each resistor wire drew approximately 1.3 A, that is, in the case of heating, a resistance wire drew 15.6 W instantaneously.

**Figure 3:**
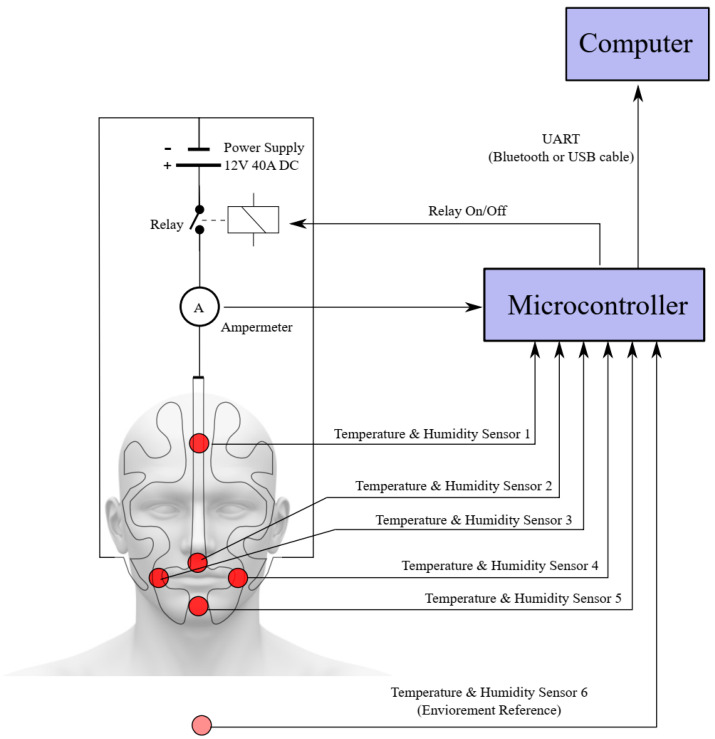
Head model wiring diagram.

The thermal head system is, basically, a heating and multi-sensor measurement system. The structure of this system is depicted in Figure 3. A control circuit was placed on the head model for the operation of the system. There were six temperature and humidity meters, one current meter, a switching unit, and a microcontroller on the circuit. The heating system in the control circuit was required to imitate body temperature, and, for this, it was necessary to keep the temperature constant, at 35 °C. For this reason, temperature control was carried out by turning the heater on and off, with the data received from a temperature sensor. In the meantime, each sensor datum was transferred to the computer via the Universal Asynchronous Transceiver (UART) of the microcontroller. DHT 22 sensor was used for temperature and relative humidity measurement. There were five temperature and relative humidity sensors in the head model. In addition, one temperature and relative humidity sensor were each reserved for ambient measurement. Depending on the average temperature of the five sensors placed on the face, the relay connected to the power source was switched on and off, and electric current was applied to the resistors. The heating process was controlled in this way. Continuous current measurement was made with the ammeter, connected in series to the resistor wires, and the measured value was both displayed on the interface and transferred to the recording file, in synchronization with the sensor data. Since the current sensor was applied at a constant voltage, it was used to calculate the applied power in Watts, by only measuring the current change. With the help of the monitoring and recording program on the computer, incoming data were processed, and a time-stamped recording report was created. Proportional Derivative Integral (PID) control, which is the most common control method, was used for temperature control. The temperature was kept constant by adjusting the opening and closing time of the relay with the applied PID control.

The water vapor, produced by a water vapor generator for the breathing system, was directed to the place where the exhalation process would take place, through the channel opened in the nose. With the breathing system, the simulation of the water vapor that comes out of the nose during breathing was provided. For the breathing system of an adult at rest, the respiratory function was performed according to the reference values specified in the literature (6 L per minute and at a respiratory frequency of 10–12 breaths). Breath control was based on the formation of water vapor, which was made by vibrations on and off the nebulizer system for a short time. The breathing process continued through the process of opening and closing periodically.

The surface and ambient temperature values (°C), obtained using the thermal head system and the relative humidity values of the microclimate and ambient air (%), were used to calculate the thermal and water vapor resistance values of the masks examined. In addition, the heat energy consumed for heating the head (W) was also a necessary parameter for the calculations. The thermal resistance (R_ct_) value was calculated according to the parallel method (surface-area-averaged resistance calculation) as shown in Equation (1) (ISO 15831), as follows:

(1) $$R_{\mathrm{ct}}=\frac{A\left(T_{s}-T_{a}\right)}{H_{c}}$$

A: total surface area of the head model (m^2^).

T_s_: average head surface temperature (K).

T_a_: average ambient temperature (K).

H_c_: The amount of heat supplied to the head model by the heater (W).

Thermal resistance measurements were carried out at ambient conditions of 20 °C and 65% relative humidity. In these ambient conditions, provided by the climate cabinet, the surface temperature of the thermal head was kept constant, at 35 °C, and the amount of power consumed to achieve this was measured during the experiment.

During the water vapor resistance measurements, both the head surface temperature and the ambient temperature were set at 35 °C. By setting the head and ambient air temperature values to a constant 35 °C, the conduction of heat transfer in the environment was prevented. The water vapor resistance of the tested masks (R_et_) was calculated using Equation (2), as stated below [25]:

(2) $$R_{\mathrm{et}}=\frac{A\left(P_{s}-P_{a}\right)}{H_{e}}$$

A: total surface area of the head model (m^2^).

P_s_: saturated water vapor pressure at head surface temperature (Pa).

P_a_: saturated water vapor pressure of ambient air (Pa).

H_e_: the amount of heat dissipated to keep the surface temperature of the head constant (W).

Partial vapor pressure values were calculated by using the saturated vapor pressure tables given, depending on the temperature [26].

In order to only obtain the values for the masks, the head was first measured bare, and the obtained measurement values were subtracted from the measurement values obtained with the mask for both thermal resistance (R_ct_) and water vapor resistance (R_et_) measurements.

**Figure 4:**
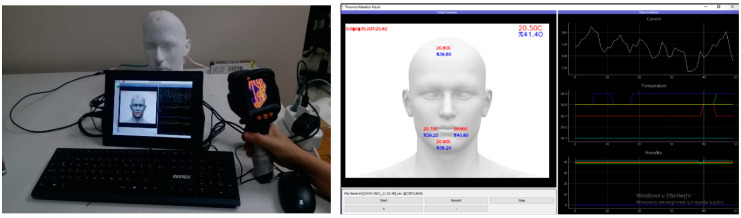
The images of the thermal head model measurement system and graphical user interface.

The thermal head model, developed in this way, has now been turned into a portable measuring device, with a heating system, breathing system, and measurement system. Thanks to this head model being made separately, products such as hats, berets, and masks will be modular parts of the manikin system, which can be measured in different environments (in the climate cabinet when necessary), using the portable head model. Images of the final head model are given in Figure 4. In order to carry out mask experiments, the thermal head model was taken into the climate cabinet. With the help of the fan placed in the climate cabinet, in accordance with ISO 11092, an air velocity of 0.5 ± 0.05 m/s for different masks in similar periods was created at the head level of the manikin. After the thermal head was tested naked, each mask was placed on the thermal head model and tested for 5 min. The system takes three measurements per second. During this period in the study, the body temperature of the thermal head was kept constant at 35 °C and the amount of power consumed to achieve this was measured.

Whether the objective data obtained showed statistically significant differences for different masks was analyzed with a one-way ANOVA test, using Duncan’s multi-distribution test. In addition, reliability analysis was also carried out to test the repeatability of the measurements performed by the thermal head system. The Cronbach Alpha technique was used to measure the internal consistency of test copies. Furthermore, varying groups according to mask types were determined by a multiple comparison Student–Newman–Kuels (SNK) post hoc test.

## 3. Results and Discussion

In this study, the thermal comfort properties of 10 different masks, which were widely used during the COVID-19 pandemic period, were examined with the thermal head model, which was developed especially for this study. For this, thermal resistance and water vapor resistance measurements were carried out when the thermal head model was naked and wearing 10 different masks.

Validation tests were carried out to analyze whether the thermal resistance and water vapor resistance measurement results of the thermal head measurement system are consistent and reproducible. A CM (cloth mask) was used as a validation mask. The validation tests were performed in two sets, for five thermal resistance and water vapor resistance measurements, each containing five trials. Of the 50 validation mask samples that were used, 25 were tested in the first set and the remaining 25 samples were then tested in the second set. According to the analysis of variance, there was no significant difference between the two sets (*p* > 0.05) and this result confirms the accuracy of the test device.

Reliability analyses were performed to evaluate the internal consistency of the measurements and the repeatability of the thermal head measurement system. Intraclass correlation coefficients, obtained by a reliability analysis, are given in Table 2. As seen in Table 2, the intraclass correlation values exceed the recommended value of 0.7, and this result shows acceptable internal consistency and reliability [27]. According to this analysis, it can be said that the thermal head measurement system gives similar and reliable results for test repetitions of the same mask.

In order to understand the ability of the thermal head measurement system to determine the differences between the masks, variance analyses were performed for the thermal resistance and water vapor resistance values. As seen in Table 3, the differences between the masks are statistically significant, meaning that the device determines the thermal resistance and water vapor resistance differences of the masks (*p* < 0.05).

**Figure 5:**
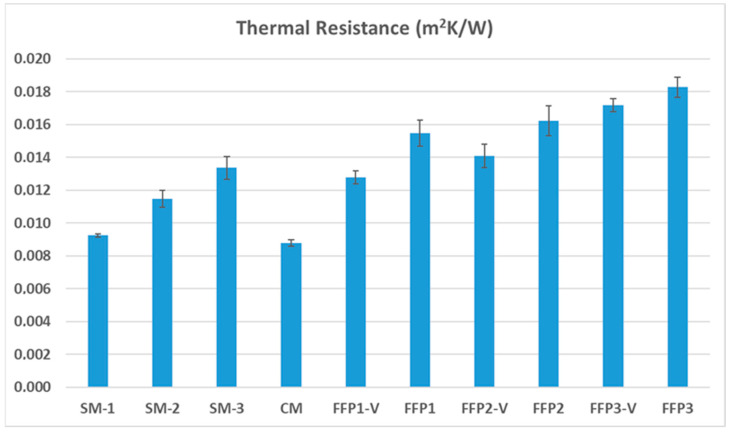
Thermal resistance results of the masks used in the study.

After ensuring the consistency, validity, reliability, and reproducibility of the results related to the thermal head measurement system, the thermal resistance and water vapor resistance properties of the masks were tested. The thermal resistance measurement results of the masks are given in Figure 5.

As seen in the thermal resistance measurement results, the lowest thermal resistance value was found for the cloth mask. As it is known in the literature, the most important parameter affecting the thermal resistance of masks is the thickness [28,29,30,31,32,33]. Although it has a higher thickness value than other mask types, the cloth mask has a fabric structure, which provides a lower thermal resistance value, compared to other non-woven mask types. It is thought that the pores formed between the warp and weft yarns allow more heat transfer than non-woven masks, which have a greater surface covering. Furthermore, as Raeisian et al. stated, the bulkier structure of non-woven masks, compared to cloth masks, causes them to give higher thermal resistance [34]. Surgical mask types generally provided lower thermal resistance than FFP masks. Among themselves, it is seen that the thermal resistance values increase as the mask ply increases. It is known from the literature that multi-ply structures provide more thermal resistance than single-ply structures [35,36]. When the results of the FFP masks are considered, it is seen that the thermal resistance values decrease due to the relatively higher transfer of the air from the inside to the outside when the valve is used in the mask structure. Thanks to the valve structure, the heated air inside goes out and reduces the microclimate temperature. Among the measured thermal resistance values, the results of the SNK post hoc test, which was applied according to mask type, are illustrated in Table 4.

The SNK post hoc test results show that the main differentiation among the effects of the different mask types was provided by the FFP3 masks, with the highest values, and cloth masks and surgical masks, with the lowest values. In addition, it should be noted that FFP masks are in the same sub-group, which means that they have a similar effect on the thermal resistance.

**Figure 6:**
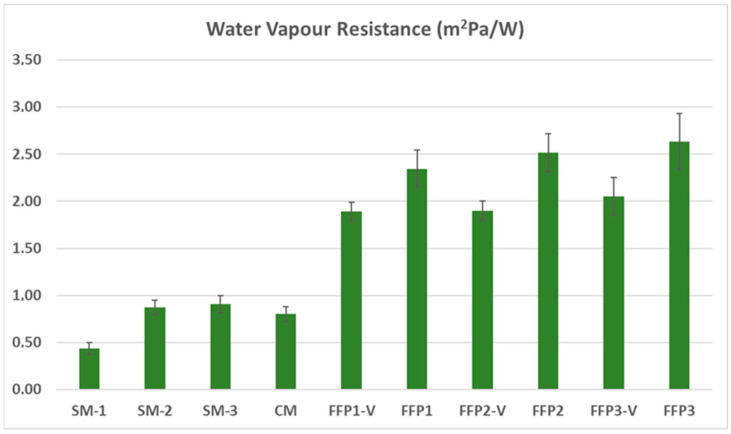
Water vapor resistance results of the masks used in the study.

For the water vapor resistance measurements of the masks, the measurement values of the temperature and the relative humidity sensors around the lips in the area covered by the mask, were effective. The water vapor resistance results are given in Figure 6.

As seen in the measurement results, the differences between the water vapor resistance values of the masks are more pronounced than the thermal resistance differences, thanks to the breathing system. While the single-ply surgical mask had the lowest water vapor resistance value, the FFP3 mask had the highest water vapor resistance value. The water vapor resistance is the most important parameter that determines the thermal comfort of the person when sweating conditions are taken into account [37]. On the other hand, when the measurement results are examined, it is noteworthy that the water vapor resistance values increase with the increase in the protection levels of the masks. As the protection level of the masks increases, the risk of aerosol contamination decreases, but the thermal comfort also decreases. Among the different masks, the cloth masks generally showed a lower water vapor resistance value. In addition, the presence of a valve in FFP masks gave lower water vapor resistance values than the valveless ones, as it allows the water vapor inside to be discharged together with the air. The analysis results of the SNK post hoc test, which was applied for mask type, are shown in Table 5.

The SNK post hoc test results show that single-ply surgical masks are separated from the other masks by being included in the first sub-group, with the lowest strength value. Other surgical masks and cloth masks were included in the second sub-group. FFP valve masks are located in the same group, and FFP valveless masks are located in the same sub-group, with the highest values.

## 4. Conclusions

In this research, the novel thermal head system was introduced. It allows measurements to be taken in different ambient conditions and allows instantaneous temperature and relative humidity measurements, to objectively evaluate the thermal resistance and water vapor resistance properties, which are the most important criteria for understanding the comfort environment created by the different mask types frequently used in COVID-19 pandemic conditions.

The thermal head measurement system that was developed in this research study differs from other devices, with its modern and technological structure; sensitive measurement capability; practical and reconfigurable 3D printing technology; effective design; simulated, real human head measurements; interface; and instant measurement capability. In light of the findings, it was observed that the thermal head measurement system provides consistent, valid, reliable, and repeatable thermal comfort measurement results.

Masks with high thermal resistance and water vapor resistance will make people feel uncomfortable during use, causing wet sweating in the mouth and nose areas, which will disturb them. For this reason, in addition to the protection of the masks, the thermal comfort levels should also be considered, especially in long-term-use cases. When the findings obtained in the study were evaluated, it was understood that the cloth masks would provide a more comfortable environment, with lower thermal resistance and water vapor resistance values, compared to the other masks. In addition, it was observed that the surgical masks offer better thermal comfort conditions, although they have lower protection than FFP masks. Among the FFP masks, which are known to have the best protection, it is noteworthy that FFP3 masks provided the most uncomfortable conditions, with the highest thermal resistance and water vapor resistance values.

The findings revealed as a result of this study may be evaluated together with the support of the medical literature studies that have measured the protection levels of masks. It is expected that the thermal head measurement system and the mask comfort data obtained as a result of this comprehensive study will contribute to the studies that have been conducted and that will be conducted as the COVID-19 pandemic continues, and will shed light on researchers and industrialists developing products in this field.

Further studies to examine the antimicrobial properties and to analyze the bacterial activities of the different mask types have been planned. Researching the hygiene and usage conditions of masks produced by different techniques may be particularly interesting, alongside an examination of their thermal comfort properties.

Since almost all models produced worldwide are manufactured as rigid surfaces, the effect of small gaps on the fit of the masks to the proposed thermal head model is assumed to be negligible. Although the process of making a human-like manikin has achieved the desired goals, in terms of electronics and software, the fact that it is made of a mechanically rigid material has limitations in terms of the results. Although the mechanical structure of the manikin is natural in terms of its shape and dimensions, it cannot fully mimic the human tissue, skin, and skeletal system. This leads to limitations in terms of the fit of the mask to the head. With the technological capabilities of 3D printing, it will be possible to use soft, flexible, and hard materials together in the future, to better imitate the human body.

## Figures and Tables

**Table 1:** Types of masks used in the study.

Sample Code	Mask Type	Thickness (mm)	View
SM-1	Single-ply surgical mask	0.36	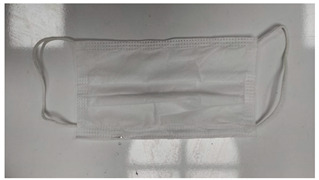
SM-2	Double-ply surgical mask	1.29	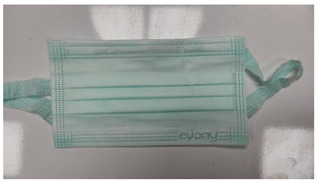
SM-3	Three-ply surgical mask	1.50	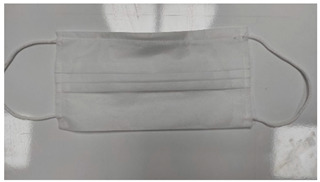
CM	Cloth mask	2.47	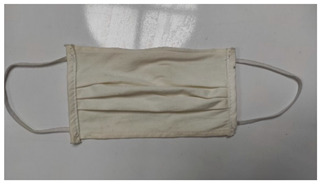
FFP1-V	FFP1 valve mask	1.41	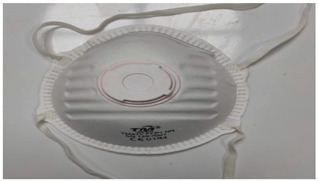
FFP1	FFP1 valveless mask	1.67	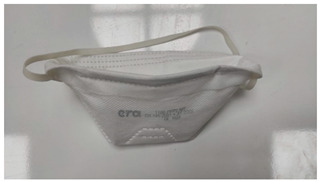
FFP2-V	FFP2 valve mask	1.41	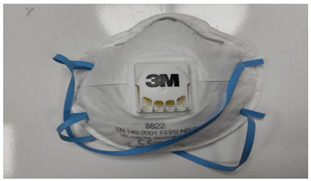
FFP2	FFP2 valveless mask	1.07	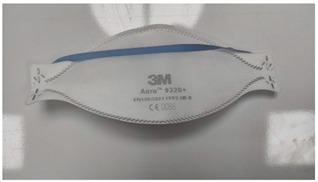
FFP3-V	FFP3 valve mask	1.54	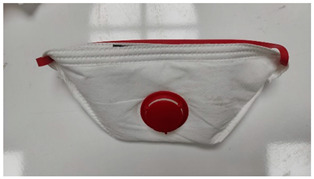
FFP3	FFP3 valveless mask	1.36	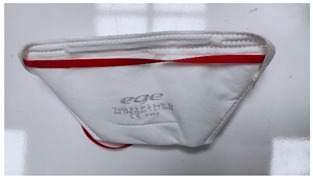

**Table 2:** The intraclass correlation coefficients obtained by reliability analysis.

Intraclass Correlation	R_ct_ Value	R_et_ Value
Single measures of intraclass correlation	0.822	0.744
Average measures of intraclass correlation	0.792	0.855

**Table 3:** The results of variance analysis for masks tested on thermal head system.

Source	Dependent Variable	Type III Sum of Squares	df	Mean Square	F	Significance
Mask Type	R_ct_	0.000	9	0.000	76.753	0.000
	R_et_	17.496	9	1.944	21576.524	0.000

**Table 4:** The results of SNK post hoc tests of the thermal resistance values.

Fabric	*n*	Subset for α = 0.05					
		1	2	3	4		
CM	25	0.0088					
SM-1	25		0.0093				
SM-2	25			0.0115			
FFP1-V	25				0.0128		
SM-3	25				0.0134		
FFP2-V	25				0.0141		
FFP1	25					0.0155	
FFP2	25					0.0162	
FFP3-V	25					0.0172	
FFP3	25						0.0183
Sig.		1.000	1.000	1.000	0.505	0.584	1.000

**Table 5:** The results of SNK post hoc tests of the water vapor resistance values.

Fabric	*n*	Subset for α = 0.05			
		1	2	3	4
SM-1	25	0.4328			
CM	25		0.8009		
SM-2	25		0.8726		
SM-3	25		0.9057		
FFP1-V	25			1.8889	
FFP2-V	25			1.9001	
FFP3-V	25			2.0546	
FFP1	25				2.3451
FFP2	25				2.5156
FFP3	25				2.6346
Sig.		1.000	0.141	0.384	0.211

## Data Availability

Not applicable.

## References

[1] WHO Director-General’s Opening Remarks at the Media Briefing on COVID-19—11 March 2020. https://www.who.int/director-general/speeches/detail/who-director-general-s-opening-remarks-at-the-media-briefing-on-covid-19---11-march-2020.

[2] Adhikari S.P., Meng S., Wu Y.-J., Mao Y.-P., Ye R.-X., Wang Q.-Z., Sun C., Sylvia S., Rozelle S., Raat H. (2020). Epidemiology, Causes, Clinical Manifestation and Diagnosis, Prevention and Control of Coronavirus Disease (COVID-19) during the Early Outbreak Period: A Scoping Review. Infect. Dis. Poverty.

[3] Nicola M., O’Neill N., Sohrabi C., Khan M., Agha M., Agha R. (2020). Evidence Based Management Guideline for the COVID-19 Pandemic-Review Article. Int. J. Surg..

[4] Weekly Epidemiological Update on COVID-19—13 July 2022. https://www.who.int/publications/m/item/weekly-epidemiological-update-on-covid-19---13-july-2022.

[5] Afroj S., Britnell L., Hasan T., Andreeva D.V., Novoselov K.S., Karim N. (2021). Graphene-Based Technologies for Tackling COVID-19 and Future Pandemics. Adv. Funct. Mater..

[6] Uddin M.A., Afroj S., Hasan T., Carr C., Novoselov K.S., Karim N. (2022). Environmental Impacts of Personal Protective Clothing Used to Combat COVID-19. Adv. Sustain. Syst..

[7] Islam M.H., Islam M.R., Dulal M., Afroj S., Karim N. (2022). The Effect of Surface Treatments and Graphene-Based Modifications on Mechanical Properties of Natural Jute Fiber Composites: A Review. iScience.

[8] Ollila H.M., Partinen M., Koskela J., Savolainen R., Rotkirch A., Laine L.T. (2021). Face Masks to Prevent Transmission of Respiratory Diseases: Systematic Review and Meta-Analysis of Randomized Controlled Trials. medRxiv.

[9] Eikenberry S.E., Mancuso M., Iboi E., Phan T., Eikenberry K., Kuang Y., Kostelich E., Gumel A.B. (2020). To Mask or Not to Mask: Modeling the Potential for Face Mask Use by the General Public to Curtail the COVID-19 Pandemic. Infect. Dis. Model..

[10] Lepelletier D., Grandbastien B., Romano-Bertrand S., Aho S., Chidiac C., Géhanno J.-F., Chauvin F. (2020). What Face Mask for What Use in the Context of the COVID-19 Pandemic? The French Guidelines. J. Hosp. Infect..

[11] Feng S., Shen C., Xia N., Song W., Fan M., Cowling B.J. (2020). Rational Use of Face Masks in the COVID-19 Pandemic. Lancet Respir. Med..

[12] Worby C.J., Chang H.-H. (2020). Face Mask Use in the General Population and Optimal Resource Allocation during the COVID-19 Pandemic. Nat. Commun..

[13] Wang C., Chudzicka-Czupała A., Grabowski D., Pan R., Adamus K., Wan X., Hetnał M., Tan Y., Olszewska-Guizzo A., Xu L. (2020). The Association Between Physical and Mental Health and Face Mask Use During the COVID-19 Pandemic: A Comparison of Two Countries With Different Views and Practices. Front. Psychiatry.

[14] Greenhalgh T., Schmid M.B., Czypionka T., Bassler D., Gruer L. (2020). Face Masks for the Public during the COVID-19 Crisis. BMJ.

[15] Sickbert-Bennett E.E., Samet J.M., Clapp P.W., Chen H., Berntsen J., Zeman K.L., Tong H., Weber D.J., Bennett W.D. (2020). Filtration Efficiency of Hospital Face Mask Alternatives Available for Use During the COVID-19 Pandemic. JAMA Intern. Med..

[16] Matuschek C., Moll F., Fangerau H., Fischer J.C., Zänker K., van Griensven M., Schneider M., Kindgen-Milles D., Knoefel W.T., Lichtenberg A. (2020). The History and Value of Face Masks. Eur. J. Med. Res..

[17] Yoon H.N., Buckley A. (1984). Improved Comfort Polyester: Part I: Transport Properties and Thermal Comfort of Polyester/Cotton Blend Fabrics. Text. Res. J..

[18] Song G. (2011). Improving Comfort in Clothing.

[19] Tang T., Zhu Y., Zhou X., Guo Z., Mao Y., Jiang H., Fang Z., Zheng Z., Chen X. (2022). Investigation of the Effects of Face Masks on Thermal Comfort in Guangzhou, China. Build. Environ..

[20] Maniaci A., Ferlito S., Bubbico L., Ledda C., Rapisarda V., Iannella G., La Mantia I., Grillo C., Vicini C., Privitera E. (2021). Comfort Rules for Face Masks among Healthcare Workers during COVID-19 Spread. Ann. Ig..

[21] Yam D., Meng T.G., Mun W., Liang W., Lee J., Teoh O.H., Rajgor D.D. (2019). A Randomised Clinical Trial to Evaluate the Safety, Fit, Comfort of a Novel N95 Mask in Children. Sci. Rep..

[22] Eberhart M., Orthaber S., Kerbl R. (2021). The Impact of Face Masks on Children—A Mini Review. Acta Paediatr..

[23] Gericke A., Militký J., Venkataraman M., Steyn H., Vermaas J. (2022). The Effect of Mask Style and Fabric Selection on the Comfort Properties of Fabric Masks. Materials.

[24] Zender-Świercz E., Telejko M., Galiszewska B. (2021). Influence of Masks Protecting against SARS-CoV-2 on Thermal Comfort. Energies.

[25] Wang F.-M., Gao C.-S., Kuklane K., Holmér I. (2009). A Study on Evaporative Resistances of Two Skins Designed for Thermal Manikin Tore under Different Environmental Conditions. J. Fiber Bioeng. Inform..

[26] ASHRAE (2017). Chapter 9, Thermal Comfort. ASHRAE Handbook—Fundamentals 2017.

[27] Black W.C., Babin B.J., Anderson R.E., Tatham R.L. (2005). Multivariate Data Analysis: Global Edition.

[28] Mandal S., Song G., Ackerman M., Paskaluk S., Gholamreza F. (2013). Characterization of Textile Fabrics under Various Thermal Exposures. Text. Res. J..

[29] Mitra A., Majumdar A., Majumdar P.K., Bannerjee D. (2013). Predicting Thermal Resistance of Cotton Fabrics by Artificial Neural Network Model. Exp. Therm. Fluid Sci..

[30] Oner E. (2019). Mechanical and Thermal Properties of Knitted Fabrics Produced from Various Fiber Types. Fibers Polym..

[31] Oglakcioglu N., Celik P., Ute T.B., Marmarali A., Kadoglu H. (2009). Thermal Comfort Properties of Angora Rabbit/Cotton Fiber Blended Knitted Fabrics. Text. Res. J..

[32] Mukhopadhyay A., Ishtiaque S.M., Uttam D. (2011). Impact of Structural Variations in Hollow Yarn on Heat and Moisture Transport Properties of Fabrics. J. Text. Inst..

[33] Mansoor T., Hes L., Bajzik V., Noman M.T. (2020). Novel Method on Thermal Resistance Prediction and Thermo-Physiological Comfort of Socks in a Wet State. Text. Res. J..

[34] Raeisian L., Mansoori Z., Hosseini-Abardeh R., Bagherzadeh R. (2013). An Investigation in Structural Parameters of Needle-Punched Nonwoven Fabrics on Their Thermal Insulation Property. Fibers Polym..

[35] Hes L., De Araujo M., Djulay V.V. (1996). Effect of Mutual Bonding of Textile Layers on Thermal Insulation and Thermal Contact Properties of Fabric Assemblies. Text. Res. J..

[36] Shabaridharan, Das A. (2012). Study on Heat and Moisture Vapour Transmission Characteristics through Multilayered Fabric Ensembles. Fibers Polym..

[37] Sampath M.B., Aruputharaj A., Senthilkumar M., Nalankilli G. (2012). Analysis of Thermal Comfort Characteristics of Moisture Management Finished Knitted Fabrics Made from Different Yarns. J. Ind. Text..

